# Onset about isothermal flow of Carreau liquid over converging channel with Cattaneo-Christov heat and mass fluxes

**DOI:** 10.1016/j.heliyon.2023.e15710

**Published:** 2023-05-03

**Authors:** Sohail Rehman, Ayman Alfaleh, Kallekh Afef, Hashim, Syed Inayat Ali Shah

**Affiliations:** a Department of Mathematics, Islamia College Peshawar, Peshawar, Pakistan; b College of Engineering, Industrial Engineering Department, Umm Al-Qura University, Al-Khalidiya District, Al-Qunfudhah City, 28821, Saudi Arabia; c Faculty of Science and Arts, King Khalid University, Mohail Asser, Saudi Arabia; d Department of Mathematics & Statistics, The University of Haripur, Haripur, 22620, Pakistan

**Keywords:** Non-Newtonian fluid, Convergent/divergent channel, Cattaneo–Christov model, Heat and mass transfer, Numerical solution

## Abstract

In this paper, an innovative mathematical approach is adopted to construct new formulation for exploring thermal characteristics in Jeffery Hamel flow between non-parallel convergent-divergent channels using non-Fourier's law. Due to the occurrence of isothermal flow of non-Newtonian fluids through non-uniform surfaces in many industrial and technological processes, such as film condensation, plastic sheet deformation, crystallization, cooling of metallic sheets, design of nozzles devices, supersonic and various heat exchangers, and glass and polymer industries, the current research is focused on this topic. To modulate this flow, the flow stream is subjected in a non-uniform channel. By incorporating relaxations in Fourier's law, thermal and concentration flux intensities are examined. In the process of mathematically simulating the flow problem, we constructed a set of governing partial differential equations that were embedded with a variety of various parameters. These equations are simplified into order differential equations using the vogue variable conversion approach. By selecting the default tolerance, the MATLAB solver bvp4c completes the numerical simulation. The temperature and concentration profiles were determined to be affected in opposing ways by thermal and concentration relaxations, while thermophoresis improved both fluxes. Inertial forces in a convergent channel accelerate the fluid in a convergent channel, while in the divergent channel the stream is shrink. The temperature distribution of Fourier's law is stronger than that of the non-Fourier's heat flux model. The study has real-world significance in the food business and is pertinent to energy systems, biomedical technology, and contemporary aircraft systems.

## Nomenclature

(r,ψ,z): Cylindrical coordinates
u(r,ψ): Fluid velocity ($ms^{-1}$)
$c_{p}$: Specific heat ($j{kg}^{-1}K^{-1}$)
γ: Channel angle
U: Maximum velocity ($ms^{-1}$)
τ: Cauchy stress tensor
T: Fluid temperature (K)
C: Concentration of fluid ($kgm^{-3}$)
$T_{w}$: Wall temperature (K)
$T_{0}$: Reference temperature (K)
$C_{w}$: Fluid concentration at the wall
μ: Generalized viscosity
$\dot{\gamma }$: Shear rate
$\mu_{0}$: Zero shear-rate viscosity
$\mu_{\infty }$: Infinite shear-rate viscosity
Γ: Material parameter of Carreau fluid
n: Power-law index of Carreau fluid
$A_{1}$: First Rivlin-Erickson tensor
p: Fluid pressure (Pa)
I: Identity tensor
$\rho_{f}$: Fluid's density ($kgm^{-3}$)
$\rho_{p}$: Nanoparticles density ($kgm^{-3}$)
$D_{T}$: Thermophoresis diffusion coefficient
$D_{B}$: Brownian diffusion coefficient
q: Heat flux
$\lambda_{C}$: Mass concentration relaxation factor
$k_{f}$: Thermal conductivity
$\nu_{f}$: Kinematic viscosity $\left(m^{2}s^{-1}\right)$
η: Dimensionless angle
$\lambda_{E}$: Thermal relaxation factor
G(η): Dimensionless velocity
θ(η): Dimensionless temperature
$\varphi \left(\eta \right)$: Dimensionless concentration
$\tau_{r\theta }$: Wall shear stress
Pr: Prandtl number
Re: Reynold number
Wi: Weissenberg number
Ec: Eckert number
Nb: Brownian motion parameter
Nt: Thermophoresis parameter
$\delta_{E}$: Thermal relaxation
$\delta_{C}$: Solutal relaxation
$C_{f}$: Skin-friction coefficient

## 1 Introduction

Heat transportation has been widely modeled utilizing energy equations based on the first law of thermodynamics, which make use of Fourier's law of heat conduction. Classical heat equations cannot be utilized to represent heat transport in polymers, where relaxation time plays a significant role in heat transport, irrespective of the fact that the conventional principle of heat conduction (Fourier's law) does not illuminate the properties of thermal time relaxation. This mechanism is essential for a variety of processes, including the termination of milk and the development of microchips and electrical devices. In traditional models of energy and mass transport (Fourier and Fick's law), the anomaly of heat and mass diffusion is disregarded. First, the idea of heat transmission was introduced by Fourier [1], and Cattaneo [2] established the concept of thermal relaxation time to designate thermal inertia. Cattaneo's model was later supplemented by Christov [3] with a time-derivative model known as the Cattaneo-Christov heat flow model. Subsequently, by considering the upper-convected derivative of Oldroyd's model, Christov [3] gave a material invariant formulation of the Cattaneo framework. To overcome the executive problem, he addresses a single energy equation. This joint modeling was named the Cattaneo-Christov model. Straughan [4] explored thermal convection in an incompressible viscous fluid by considering the Cattaneo-Christov model. Ciarletta and Straughan [5] discussed the structural stability and validity of the Cattaneo-Christov equations. Han et al. [6] examined the slip flow and heat transport of a viscoelastic fluid bounded by a stretching plate using the Cattaneo-Christov law. Mustafa [7] provided an exact and approximate solution for the rotating flow of Maxwell's fluid by considering the Cattaneo-Christov model. Tibullo and Zampoli [8] investigated the behavior of the Cattaneo–Christov form when coupled to incompressible liquids, yielding unique results in the procedure. Hayat et al. [9] used the homotopic analysis technique to extend an analytic solution for the flow through a diverse thickness sheet in addition to Cattaneo–Christov heat flux model. The impact of a non-uniform heat source and sink on the three-dimensional magnetohydrodynamic was investigated by Ramadevi et al. [10] considering modified Fourier's law, Carreau fluid flow over a stretched surface. The Cattaneo-Christove law of heat flow was utilized by Nazir et al. [11] to get the heat equation, which was then applied to the formulation of transport in the Carreau fluid, and difficulties were then resolved using FEM. In a micropolar fluid, Nawaz et al. [12] investigated the simultaneous impacts of nanomaterials, thermal relaxation time, and spin gradient viscosity on heat transmission.

Nanofluid has several uses in a variety of industries, including medicine, manufacturing, microfluidics, transportation, microelectronics, and energy conservation. Problems with heat transformation affect each of these components. The nanofluids increase the rate of heat transfer, cut down on time-consuming activities, and improve the effectiveness of mechanisms. Under the influence of dynamism of force, the fluid not only causes the transmission of momentum but also the depletion and transfer of mass and energy. Heat transfer is the primary physical manifestation of energy transmission. Nanofluids have received a lot of attention in the disciplines of engineering and biology due to their outstanding thermal conductivity. Under specific presumptions, Buongiorno [13] proposed a nanofluids convective transport model in 2006, which was referred to as the Buongiorno model. He elaborated on the concepts of thermophoresis and Brownian diffusion as well as the formulation of the leading equations for momentum, temperature, and concentration of viscous nanofluids. A significant contribution in science and biology is provided by the Brownian motion (BM), a random particle motion. The constant barrage of chemicals in the environment's media results in BM. This type of motion results from contact with nearby liquid or gas molecules. Large structural molecules move in response to a macroscopic temperature difference through a process called thermophoresis. The presentation of numerous particle reactions is what triggers the phenomena. When it comes to issues with heat and mass transfer, these Brownian and thermophoresis (BMT) processes are essential. Numerous scholars recently investigated how distinct nanofluid streams were affected by Brownian and thermophoretic diffusions. The joint effects of thermophoresis, Brownian motion, and nanoparticles on the transport mechanism in the 3-dimensional flow of Maxwell fluid were examined by Sreedevi and Reddy [14]. Many researchers [6,8,15,16] have used the Cattaneo-Christov double diffusion or the Buongiorno model alone to study the transfer of heat and mass through fluids. In order to generate thermophoretic and Brownian parameters with relaxation effect, the Buongiorno model is taken into consideration. However, the model does not substantially use double diffusive Cattaneo-Christov to rectify the Brownian diffusion and thermal conductivity factor.

The convergent-divergent channel flows have extensive application in mechanical, biomechanical, civil, chemical, aeronautical, and industrial engineering. Additionally, there are many ways to use this mathematical model to study how rivers and canals flow as well as how blood flows within the human body. Therefore, it is vital to comprehend the flow in this type of channel in order to address technical challenges [[17], [18], [19], [20]]. The radial 2D incompressible flow of a viscous fluid through convergent or divergent channels was first developed by Jeffery [21] in 1915. Since the famed Jeffery-Hamel flow is widely recognized as a component of the one-of-a-kind accurate solution to the Navier-Stokes equation, it is of utmost significance. Flows across convergent-divergent channels have drawn a lot of attention from researchers due to their significant significance for many engineering applications. Khan et al. [22] inquired Dufour and Soret impact of second-grade fluid in the context of Jeffery-Hamel flow in the non-uniform channel. The analytical and numerical methods were employed to solve the non-linear equations. Li et al. [23] used the Galerkin approach to measure the energy transport of Jeffery-Hamel (J-H) nanofluid flow among inclined walls by employing Brinkman and Maxwell-Garnetts models. Mahmood et al. [24] used the Spectral Homotopic Analysis method (S-HAM) to evaluate the thermal presentation of a stable 2D viscous, incompressible fluid in converging-divergent channels driven by a transversely magnetic field. In another study, Moradi et al. [25] explored the J-H flow for nanofluids and evaluated the heat transfer effect during the flow. They used the differential transformation approach and the Runge-Kutta scheme and provide exact and numerical solutions. Gerdroodbary et al. [26] investigated the thermal radiation through stretchable convergent-divergent channels in classical J-H flow. Non-Newtonian fluid flows between nonparallel walls have been studied in several various geometries due to their industrial uses. These investigations include those by Balmer and Kauzlaich [27], Forsyt [28], Chakraborty and Metzner [29], Bhatnagar et al. [30] and Baris [31].

In the light of current discussion, we conclude that no study has been witnessed to discuss the Catteno-Christov heat flux model for Carreau nanofluid flows and heat transformation along an inclined, converging diverging channel. We assume that the description of double diffusional Catteneo-Christov thermal and solutal fluxes stimulated with nanofluids discharged over a non-uniform surface. We used cylindrical polar coordinates to model the geometry of the flow because that is the best fit for the surface on which the flow is emancipated. The novelty of this research elaborates the non-Fourier's heat flux model and Fick's law due to heat and mass transfer features of non-Newtonian Carreau fluid flow in conjunction with Buongiorno nanoparticles model along converging/diverging channels. Because conventional Fourier's law is incapable to give precise estimates about heat transport in non-Newtonian liquids. The originality of this research is in the use of the Catteneo-Christov heat flux analysis for the Jeffery-Hamel flow of non-Newtonian Carreau liquid, including nanofluids, for the first time to examine a dynamical system. The momentum equation is modeled using Carreau model in radial co-ordinates. Thirdly, the numerical Bvp4c approach also known as the extended direct computational method was used to solve the governing equations. Secondly, the energy and concentration equations are modeled using modified Fourier's law and Fick's law. The modeled PDEs are transformed into a set of non-linear ordinary differential equations with the aid of similarity transformations. The numerical findings are presented graphically. The flow behavior under diverse parameters is observed. Brief analyses are executed to examine the several flow parameters in velocity, energy, and concentration fields. It is discovered that increasing the channel angle and Reynolds number in convergent flow causes an increase in the velocity profiles, indicating the exclusion of backflow, whereas a diverging flow exhibits the opposite effect. Thermal time constant also relates to the functionality of thermal elastic materials and is accountable for renovating thermal equilibrium in a fluid. The temperature region thickness is reduced because of this thermal relaxation phenomenon. The outcomes could be applied to the cooling of equipment, electronics, and various industrial units.

## 2 Model formulation

**Fig. 1:**
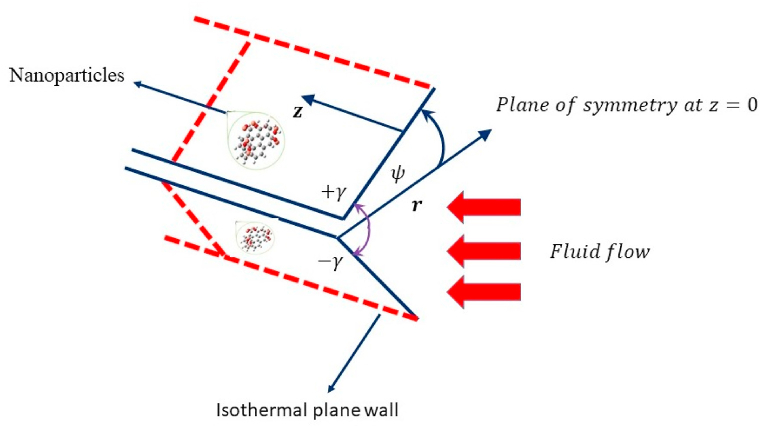
Geometric orientation of flow model.

A comprehensive analysis of steady, incompressible Jeffrey Hamel flow across non-parallel walls using the Cattaneo-Christov heat flux model with heat and mass transfer has been accomplished. The walls of the channel are inclined at an angle γ with the horizontal axis, which specifies the direction in which the walls are oriented as portrayed in Fig. 1. For γ>0 and γ<0, the walls are assumed to be diverging and converging, respectively. The flow of Carreau fluid is presumed by a non-uniform channel in the r and ψ with no change in the z-direction, r is the radial axis and ψ is the central line angle with the wall of the channel. The total angle between the channel is ψ such that −γ<ψ<γ and ψ=2γ. Furthermore, the external forces such as gravity are negligible. The velocity vector, temperature, and concentration are assumed to be $\vec{V}=\left(u\left(r,\psi \right),0,0\right)$, T(r,ψ), and C(r,ψ) respectively. The Cattaneo–Christov heat flux model is employed to scrutinize the thermal and mass transfer mechanism. Furthermore, the migration of nanoparticles results in the occurrence of two essential phenomena: thermophoresis and Brownian motion as stated by the Buongiorno model is employed. Consequently, the existence of these two forces is deemed necessary to produce realistic results. The temperature and concentration of the walls are anticipated to be constant are prescribed by $T_{w}$ and $C_{w}$, respectively.

## 3 Constitutive relation and basic equations

### 3.1 Mass conservation equation

The balance momentum and energy equation for an incompressible fluid are [32]. The conservation of mass in vector notation:

(1) $$\mathrm{div}\;\vec{V}=0\text{.}$$

In component form, the mass conservation becomes

(2) $$\frac{\partial \left(ru\left(r,\psi \right)\right)}{\partial r}=0\text{.}$$

### 3.2 Momentum equation

The conservation of momentum in vector notation free of body force is written as:

(3) $$\rho_{f}\left(V∙\nabla V\right)=\mathrm{div}\;\tau \text{.}$$

Where $\nabla =\frac{\partial }{\partial r}\hat{e_{r}}+\frac{1}{r}\frac{\partial }{\partial \psi }\hat{e_{\psi }}+\frac{\partial }{\partial z}\hat{e_{z}}$ is the gradient, and $\rho_{f}$ symbolize the fluid density, V denote the velocity field. Furthermore, τ signifies the Cauchy stress tensor for Carreau fluid [33,34]

(4) $$\tau =-pI+\mu A_{1}\text{,}$$

where

(5) $$\mu =\mu_{0}{\left[1+{\left(\Gamma \dot{\gamma }\right)}^{2}\right]}^{\frac{n-1}{2}}\text{,}$$

and

(6) $$\dot{\gamma }={\left[2{\left(\frac{\partial u\left(r,\psi \right)}{\partial r}\right)}^{2}+\frac{1}{r^{2}}{\left(\frac{\partial u\left(r,\psi \right)}{\partial \psi }\right)}^{2}+\frac{2{u\left(r,\psi \right)}^{2}}{r^{2}}\right]}^{\frac{1}{2}}\text{.}$$

After utilizing Eqs. (4), (5), (6)) in Eq. (3), the momentum equation in cylindrical polar coordinates takes the form [35]:

(7) $$u\left(r,\psi \right)\frac{\partial u\left(r,\psi \right)}{\partial r}=-\frac{1}{\rho_{f}}\frac{\partial p}{\partial r}+v_{f}\left[\frac{1}{r}\frac{\partial }{\partial r}\left\{r{\left(1+\Gamma^{2}\left(2{\left(\frac{\partial u\left(r,\psi \right)}{\partial r}\right)}^{2}+\frac{1}{r^{2}}{\left(\frac{\partial u\left(r,\psi \right)}{\partial \psi }\right)}^{2}+\frac{2{u\left(r,\psi \right)}^{2}}{r^{2}}\right)\right)}^{\frac{n-1}{2}}2\frac{\partial u}{\partial r}\right\}+\frac{1}{r}\frac{\partial }{\partial \theta }\left\{{\left(1+\Gamma^{2}\left(2{\left(\frac{\partial u\left(r,\psi \right)}{\partial r}\right)}^{2}+\frac{1}{r^{2}}{\left(\frac{\partial u\left(r,\psi \right)}{\partial \psi }\right)}^{2}+\frac{2{u\left(r,\psi \right)}^{2}}{r^{2}}\right)\right)}^{\frac{n-1}{2}}\frac{1}{r}\frac{\partial u}{\partial \theta }\right\}-\left\{{\left(1+\Gamma^{2}\left(2{\left(\frac{\partial u\left(r,\psi \right)}{\partial r}\right)}^{2}+\frac{1}{r^{2}}{\left(\frac{\partial u\left(r,\psi \right)}{\partial \psi }\right)}^{2}+\frac{2{u\left(r,\psi \right)}^{2}}{r^{2}}\right)\right)}^{\frac{n-1}{2}}\frac{2u\left(r,\psi \right)}{r}\right\}\right]\text{,}$$

(8) $$0=-\frac{1}{\rho_{f}r}\frac{\partial p}{\partial \psi }+v_{f}\left[\frac{1}{r^{2}}\frac{\partial }{\partial r}\left\{r^{2}{\left(1+\Gamma^{2}\left\{2{\left(\frac{\partial u\left(r,\psi \right)}{\partial r}\right)}^{2}+\frac{1}{r^{2}}{\left(\frac{\partial u\left(r,\psi \right)}{\partial \psi }\right)}^{2}+\frac{2{u\left(r,\psi \right)}^{2}}{r^{2}}\right\}\right)}^{\frac{n-1}{2}}\frac{1}{r}\frac{\partial u\left(r,\psi \right)}{\partial \psi }\right\}+\frac{1}{r}\frac{\partial }{\partial \theta }\left\{{\left(1+\Gamma^{2}\left(2{\left(\frac{\partial u\left(r,\psi \right)}{\partial r}\right)}^{2}+\frac{1}{r^{2}}{\left(\frac{\partial u\left(r,\psi \right)}{\partial \psi }\right)}^{2}+\frac{2{u\left(r,\psi \right)}^{2}}{r^{2}}\right)\right)}^{\frac{n-1}{2}}\frac{2u\left(r,\psi \right)}{r}\right\}\right]\text{.}$$

Integrating the equation of continuity which yield as:

(9) $$u\left(r,\psi \right)=\frac{f\left(\psi \right)}{r}\text{.}$$

After implementation of Eq. (9) and eliminating the pressure gradient from Eqs. (7), (8), we arrive at a single equation.

(10) $$\left[f^{'''}+4f^{'}\right]{\left[1+\frac{\Gamma^{2}}{r^{4}}\left(f^{'2}+4f^{2}\right)\right]}^{\frac{n-1}{2}}+\frac{2ff^{'}}{\nu_{f}}+\frac{\left(n-1\right)\Gamma^{2}}{r^{4}}{\left[1+\frac{\Gamma^{2}}{r^{4}}\left(f^{'2}+4f^{2}\right)\right]}^{\frac{n-3}{2}}\left[3f^{'}f^{''2}+32ff^{'}f^{''}+f^{'2}f^{'''}+64f^{'}f^{2}\right]+\frac{\left(n-1\right)\left(n-3\right)\Gamma^{4}}{r^{8}}{\left[1+\frac{\Gamma^{2}}{r^{4}}\left(f^{'2}+4f^{2}\right)\right]}^{\frac{n-5}{2}}\left[f^{'3}f^{''2}+16ff^{'3}f^{''}+32f^{3}f^{'}f^{''}+16f^{2}f^{'3}+64f^{4}f^{'}-4f^{'5}\right]=0$$

With associated boundary conditions [36,37].

At the channel central line

(11) $${u=U|}_{\psi =0},{\frac{\partial u}{\partial \theta }=0|}_{\psi =0}\text{.}$$

At the symmetry

(12) $$u={0|}_{\psi =\pm \gamma }$$

Introducing well-established similarity variables [36,38].

(13) $$g\left(\eta \right)=\frac{f\left(\psi \right)}{rU},\eta =\frac{\psi }{\gamma },\theta \left(\eta \right)=\frac{T}{T_{w}},\varphi \left(\eta \right)=\frac{C}{C_{w}}\text{.}$$

The usage of Eq. (13), into Eqs. (10), (11), (12), yield dimensionless ODEs with unknown velocity in combination with constraints at the boundary:

(14) $$\left[g^{'''}+4\gamma^{2}g^{'}\right]{\left[1+W_{i}^{2}\left(g^{'2}+4\gamma^{2}g^{2}\right)\right]}^{\frac{n-1}{2}}+2\gamma Regg^{'}+\left(n-1\right)W_{i}^{2}{\left[1+W_{i}^{2}\left(g^{'2}+4\gamma^{2}g^{2}\right)\right]}^{\frac{n-3}{2}}\left[3g^{'}g^{''2}+32\gamma^{2}gg^{'}g^{''}+g^{'2}g^{'''}+64\gamma^{4}g^{'}g^{2}\right]+\left(n-1\right)\left(n-3\right)W_{i}^{4}{\left[1+W_{i}^{2}\left(g^{'2}+4\gamma^{2}g^{2}\right)\right]}^{\frac{n-5}{2}}\left[g^{'3}g^{''2}+16\gamma^{2}gg^{'3}g^{''}+32\gamma^{4}g^{3}g^{'}g^{''}+16\gamma^{4}g^{2}g^{'3}+64\gamma^{4}g^{4}g^{'}-4\gamma^{2}g^{'5}\right]=0\text{,}$$

(15) $$g\left(0\right)=1,g^{\prime }\left(0\right)=0,g\left(1\right)=0\text{.}$$

Here $W_{i}^{2}=\left(\frac{\Gamma^{2}U^{2}}{r^{2}\gamma^{2}}\right),Re=\frac{\gamma rU}{v_{f}}\text{,}$ designate the dimensionless Weissenberg and Reynold number. Moreover, the n, indicate the power index, which describes the rheological behavior of Carreau non-Newtonian fluid.

### 3.3 Wall drag force

Skin friction in the view of the definition

(16) $$C_{f}={\frac{\tau_{r\theta }}{\rho_{f}U^{2}}|}_{\theta =\alpha }$$

where $\tau_{w}$ is the corresponding shear stress

(17) $$\tau_{r\theta }=\mu_{f}{\left\{{\left(1+\Gamma^{2}\left(\frac{1}{r^{2}}{\left(\frac{\partial u\left(r,\psi \right)}{\partial \psi }\right)}^{2}+2{\left(\frac{\partial u\left(r,\psi \right)}{\partial r}\right)}^{2}+\frac{2{u\left(r,\psi \right)}^{2}}{r^{2}}\right)\right)}^{\frac{n-1}{2}}\frac{2u\left(r,\psi \right)}{r}\right\}|}_{\theta =\alpha }\text{,}$$

Implementing, Eqs. (13), (17), in Eq. (16), we establish the relation for skin friction coefficient which follows as

(18) $$ReC_{f}=\left[\left[{\left(1+W_{i}^{2}\left(4\gamma^{2}g^{2}\left(1\right)+{g^{'}}^{2}\left(1\right)\right)\right)}^{\frac{n-1}{2}}\right]g^{'}\left(1\right)\right]$$

## 4 Thermal distribution analysis

Heat transfer within the confined system of converging-diverging channels is prescribed in this section. The temperature of the confined system achieved from the basic conservation energy equation in unification with the *Cattaneo*-*Christov* heat flux theory. The energy equation in conjunction with Buongiorno theory and non-Fourier model, is prescribed in following manner.

### 4.1 Energy equation

Vector form of energy equation along with nano-particles dissipative effects is established as [6]:

(19) $${\left(\rho c_{p}\right)}_{f}\left(V∙\nabla T\right)-{\left(\rho c_{p}\right)}_{f}\left[D_{B}\left(\nabla C∙\nabla T\right)+\frac{D_{T}}{T_{0}}\left({\nabla T}^{2}\right)\right]=-\nabla ∙q+\tau :\nabla V\text{.}$$

By incorporating mass and heat flux relaxations, we investigate the dual diffusion indication in viewing thermal and mass migrations. The following equations are used by the Catteneo-Christov model to define the energy diffusion [39,40]:

(20) $$q+\lambda_{E}\left[V∙\nabla q-q∙\nabla V+\left(\nabla ∙V\right)q\right]=-k_{f}\nabla T\text{,}$$

(21) $$J+\lambda_{C}\left[V∙\nabla J-J∙\nabla V+\left(\nabla ∙V\right)J\right]=-D_{B}\nabla C\text{,}$$

where (q,J) are the thermal and mass fluxes, $k_{f}$ is thermal conductivity, $\left(\lambda_{E},\lambda_{C}\right)$ denotes the thermal and mass relaxation time, $D_{B}$ is the mass diffusion coefficient. The assumed flow is incompressible therefore ∇∙V=0, the energy expression will become:

(22) $$q+\lambda_{E}\left[V∙\nabla q-q∙\nabla V\right]=-k_{f}\nabla T\text{.}$$

with $\lambda_{E}=0\text{,}$ the classical, Fourier's law $q=-k_{f}\nabla T$ is deduced. Eliminating q from Eqs. (20), (22), the energy equation with an unknown temperature T can be described as [41,42]:

(23) $$u\left(r,\psi \right)\frac{\partial T\left(r,\psi \right)}{\partial r}+\lambda_{E}\left[u\frac{\partial u\left(r,\psi \right)}{\partial r}\frac{\partial T\left(r,\psi \right)}{\partial r}+{u\left(r,\psi \right)}^{2}\frac{\partial^{2}T\left(r,\psi \right)}{\partial r^{2}}\right]+2\mu_{0}\lambda_{E}{\left[1+\Gamma^{2}\left\{2{\left(\frac{\partial u\left(r,\psi \right)}{\partial r}\right)}^{2}+\frac{1}{r^{2}}{\left(\frac{\partial u\left(r,\psi \right)}{\partial \psi }\right)}^{2}+\frac{2{u\left(r,\psi \right)}^{2}}{r^{2}}\right\}\right]}^{\frac{n-1}{2}}\left[2u\left(r,\psi \right)\left(\frac{\partial u\left(r,\psi \right)}{\partial r}\right)\frac{\partial^{2}u\left(r,\psi \right)}{\partial r^{2}}-\frac{2u\left(r,\psi \right)}{r^{3}}{\left(\frac{\partial u\left(r,\psi \right)}{\partial \psi }\right)}^{2}+\frac{u\left(r,\psi \right)}{r^{2}}\frac{\partial^{2}u\left(r,\psi \right)}{\partial r\partial \psi }\frac{\partial u\left(r,\psi \right)}{\partial \psi }+\frac{2{u\left(r,\psi \right)}^{2}}{r^{2}}\left(\frac{\partial u\left(r,\psi \right)}{\partial r}\right)-\frac{2{u\left(r,\psi \right)}^{3}}{r^{3}}\right]-\in D_{B}\lambda_{E}\left[u\left(r,\psi \right)\frac{\partial^{2}C\left(r,\psi \right)}{\partial r^{2}}\frac{\partial T\left(r,\psi \right)}{\partial r}+u\left(r,\psi \right)\frac{\partial^{2}T\left(r,\psi \right)}{\partial r^{2}}\frac{\partial C\left(r,\psi \right)}{\partial r}-\frac{2{u\left(r,\psi \right)}^{2}}{r^{3}}\frac{\partial T\left(r,\psi \right)}{\partial \psi }\frac{\partial C\left(r,\psi \right)}{\partial \psi }+u\left(r,\psi \right)\frac{\partial^{2}T\left(r,\psi \right)}{\partial r\partial \psi }\frac{\partial C\left(r,\psi \right)}{\partial \psi }+\frac{u\left(r,\psi \right)}{r^{2}}\frac{\partial^{2}C\left(r,\psi \right)}{\partial r\partial \psi }\frac{\partial T\left(r,\psi \right)}{\partial \psi }\right]-2\in \frac{D_{T}\lambda_{E}}{T_{\infty }}\left[u\left(r,\psi \right)\frac{\partial u\left(r,\psi \right)}{\partial r}\frac{\partial^{2}T\left(r,\psi \right)}{\partial r^{2}}-\frac{u\left(r,\psi \right)}{r^{3}}{\left(\frac{\partial T\left(r,\psi \right)}{\partial \psi }\right)}^{2}+\frac{u\left(r,\psi \right)}{r^{2}}\frac{\partial u\left(r,\psi \right)}{\partial \psi }\frac{\partial^{2}T\left(r,\psi \right)}{\partial r\partial \psi }\right]+{\frac{\mu_{0}}{{\left(\rho c_{p}\right)}_{f}}\left[1+\Gamma^{2}\left\{2{\left(\frac{\partial u\left(r,\psi \right)}{\partial r}\right)}^{2}+\frac{1}{r^{2}}{\left(\frac{\partial u\left(r,\psi \right)}{\partial \psi }\right)}^{2}+\frac{2{u\left(r,\psi \right)}^{2}}{r^{2}}\right\}\right]}^{\frac{n-1}{2}}\left[\left\{2{\left(\frac{\partial u\left(r,\psi \right)}{\partial r}\right)}^{2}+\frac{1}{r^{2}}{\left(\frac{\partial u\left(r,\psi \right)}{\partial \psi }\right)}^{2}+\frac{2{u\left(r,\psi \right)}^{2}}{r^{2}}\right\}\right]=\frac{k_{f}}{{\left(\rho c\right)}_{f}}\left[\frac{1}{r}\frac{\partial T\left(r,\psi \right)}{\partial r}+\frac{\partial^{2}T\left(r,\psi \right)}{\partial r^{2}}+\frac{1}{r^{2}}\frac{\partial^{2}T\left(r,\psi \right)}{\partial \psi^{2}}\right]+\in \left(D_{B}\left[\frac{\partial T\left(r,\psi \right)}{\partial r}\frac{\partial C\left(r,\psi \right)}{\partial r}+\frac{1}{r^{2}}\frac{\partial T\left(r,\psi \right)}{\partial \psi }\frac{\partial C\left(r,\psi \right)}{\partial \psi }\right]+\frac{D_{T}}{T_{\infty }}\left[{\left(\frac{\partial T\left(r,\psi \right)}{\partial r}\right)}^{2}+\frac{1}{r^{2}}{\left(\frac{\partial T\left(r,\psi \right)}{\partial \psi }\right)}^{2}\right]\right)$$

with the following constraints at the boundaries.

The temperature within the central line of the channel:

(24) $$\frac{\partial T\left(r,\psi \right)}{\partial \psi }={0|}_{\psi =0}\text{.}$$

The temperature at the walls of the channel:

(25) $${{T\left(r,\psi \right)=T}_{w}|}_{\psi =\pm \gamma }\text{.}$$

After implementing Eq. (13), into Eq. (23), the dimensionless temperature distribution equation takes the forms:

(26) $$\theta^{''}+\mathrm{Pr}\;N_{B}\theta^{'}\varphi^{'}+\mathrm{Pr}\;N_{T}\theta^{'2}+PrEc\left[{\left[\left(1+W_{i}^{2}\left(4\gamma^{2}g^{2}+{g^{'}}^{2}\right)\right)\right]}^{\frac{n-1}{2}}\right]\left(4\gamma^{2}g^{2}+{g^{'}}^{2}\right)-2\delta_{E}PrEc{\left[\left(1+W_{i}^{2}\left(4\gamma^{2}g^{2}+{g^{'}}^{2}\right)\right)\right]}^{\frac{n-1}{2}}\left[3{g^{'}}^{2}g+8\gamma^{2}g^{3}\right]-2\delta_{E}\;\mathrm{Pr}\left[N_{B}\theta^{'}\varphi^{'}g+N_{T}\theta^{'2}g\right]=0\text{,}$$

(27) $$\theta \left(1\right)=1,\theta^{\prime }\left(0\right)=0\text{.}$$

Here $\mathrm{Pr}=\frac{{\left(\mu C_{p}\right)}_{f}}{k_{f}}\text{,}$ $N_{B}=\frac{\tau D_{B}C_{w}}{\nu_{f}}$, $N_{T}=\frac{\tau D_{T}T_{w}}{\nu_{f}T_{0}}$, $Ec=\frac{U^{2}}{C_{p}T_{w}}$, $\delta_{E}=\lambda_{E}U$, $\in =\frac{{\left(\rho c_{p}\right)}_{p}}{{\left(\rho c_{p}\right)}_{f}}$ are the distinguish dimensionless parameters i.e., the Prandtl, Brownian diffusion, the thermophoresis, the Eckert thermal relaxation parameter, and the ratio of nanoparticle to the nanofluid respectively.

## 5 Concentration distribution analysis

This section presents the nanofluid concentration within the flow field subject to modified mass flux as given by Cattaneo-Christonv relation.

### 5.1 Concentration equation

Equation of Mass concentration in vector notation

(28) $$\left(V∙\nabla C\right)-\left[D_{B}\nabla^{2}C+D_{T}\frac{\nabla^{2}T}{T_{0}}\right]=-\nabla ∙J$$

Using assumption ∇∙V=0, Eq. (21) reduces to:

(29) $$J+\lambda_{C}\left[V∙\nabla J-J∙\nabla V\right]=-D_{B}\nabla C\text{.}$$

where $D_{B}$, $D_{T,}$ and $\lambda_{C}$ specify the Brownian, thermophoresis, and solution relaxation time, respectively. On eliminating J, from Eqs. (28), (29), the mass concentration equation takes the following form:

(30) $$u\left(r,\psi \right)\frac{\partial C\left(r,\psi \right)}{\partial r}+\lambda_{C}\left[u\left(r,\psi \right)\frac{\partial u\left(r,\psi \right)}{\partial r}\frac{\partial C\left(r,\psi \right)}{\partial r}+u^{2}\left(r,\psi \right)\frac{\partial^{2}C\left(r,\psi \right)}{\partial r^{2}}\right]-\frac{D_{T}}{T_{w}}\lambda_{C}\left[u\left(r,\psi \right)\frac{\partial^{3}T\left(r,\psi \right)}{\partial r^{3}}-\frac{u\left(r,\psi \right)}{r^{2}}\frac{\partial T\left(r,\psi \right)}{\partial r}+u\left(r,\psi \right)\frac{\partial^{2}T\left(r,\psi \right)}{\partial r^{2}}-\frac{2u^{2}\left(r,\psi \right)}{r^{3}}\frac{\partial^{2}T\left(r,\psi \right)}{\partial \theta^{2}}+\frac{u\left(r,\psi \right)}{r^{2}}\frac{\partial^{3}T\left(r,\psi \right)}{\partial r\partial \theta^{2}}\right]=D_{B}\left(\frac{1}{r}\frac{\partial C\left(r,\psi \right)}{\partial r}+\frac{\partial^{2}C\left(r,\psi \right)}{\partial r^{2}}+\frac{1}{r^{2}}\frac{\partial^{2}C\left(r,\psi \right)}{\partial \theta^{2}}\right)+\frac{D_{T}}{T_{w}}\left[\frac{1}{r}\frac{\partial T\left(r,\psi \right)}{\partial r}+\frac{\partial^{2}T\left(r,\psi \right)}{\partial r^{2}}+\frac{1}{r^{2}}\frac{\partial^{2}T\left(r,\psi \right)}{\partial \theta^{2}}\right]$$

The mass concentration at the central region of the channel

(31) $$\frac{\partial C\left(r,\psi \right)}{\partial \psi }={0|}_{\psi =0}\text{.}$$

At the wall

(32) $${{C\left(r,\psi \right)=C}_{w}|}_{\psi =\pm \gamma }\text{,}$$

Usage of Eq. (13) into Eq. (30), we obtain,

(33) $$\varphi^{\prime \prime }+\frac{N_{T}}{N_{B}}\theta^{\prime \prime }-2\delta_{c}\frac{N_{T}}{N_{B}}{g\theta }^{\prime \prime }=0\text{,}$$

(34) $$\varphi \left(1\right)=1,\varphi^{\prime }\left(0\right)=0\text{.}$$

## 6 Solution methodology and verification

The MATLAB bvp4c procedure is a simple and user-friendly tool that can handle challenging problems. The administrative Eqs. (15), (26), (33) with associated boundary conditions are tickled via an efficient Shooting algorithm coupled with BVP4c procedures with step size Δη=0.0001. The system of a dimensionless ordinary differential equation is transformed into first order using new variables. The numerical solutions are carried out by MATLAB built-in function BVP4c solver [43]. Assume that the range of integration is of finite dimension by taking $\eta_{\max}=10\text{.}$ A brief comparison is taken out between the calculated values of g(η), θ(η,) and φ(η) with the boundary conditions $g^{\prime }\left(\eta_{\max}\right)=1$, $\theta \;\left(\eta_{\max}\right)=1$ and $\varphi \left(\eta_{\max}\right)=1$. The numerical outcomes are for flow, fluid temperature, concentration, and skin friction are under the action of various physical parameters are depicted and discussed in detail.

### 6.1 Verification

It is impossible to make comparisons with experimental data because they are not available. To contrast our results with previous findings, more straightforward investigation, we have included a comparison. Comparisons between the Bvp4c code for velocity g(η), skin friction ${ReC}_{f}=g^{\prime }\left(1\right)$ and Nusselt number $-\frac{1}{\gamma }\theta^{'}\left(1\right)$ with published findings are shown in Table 1, Table 2, Table 3, respectively, for the special reduced ($W_{i}=0$) or (n=0) (Newtonian fluid) scenario. It is possible to get excellent correlation between the stream function g(η) skin friction ${ReC}_{f}=g^{\prime }\left(1\right)$ and Nusselt number ($\delta_{E}=0$) at Reynolds numbers and vertex angle of the divergent/converging channel γ. It should be emphasized that the findings confirm that there is a great match in the numerical data.

**Table 1:** Comparison of current values of velocity g(η) with existing literature in a limiting case when, Re=50, $\gamma =3^{0}$, $W_{i}=0$, n=1.

η	g(η)		g(η)	
	[44]	Current model outcomes	[44]	Current model outcomes
−1.00	0	0	0	0
– 0.75	0.346178919450240	0.3417	0.95686192461042	0.9586
– 0.50	0.66932017411666	0.6692	0.81145737694425	0.8114
– 0.25	0.90976534274089	0.9097	0.51582799527763	0.5158
0.00	1.000000000	1.0000	1.000000000	1.0000
0.25	0.90976534274089	0.9098	0.51582799527763	0.5157
0.50	0.66932017411666	0.6693	0.81145737694425	0.8114
0.75	0.346178916274248	0.3461	0.95686192461042	0.9589
1.00	0.0000000000	0.0000	0.000000000	0.0000

**Table 2:** Comparison of existing values of skin friction ${ReC}_{f}=g^{\prime }\left(1\right)$ with earlier results without for $\gamma =5^{0}$ and $\gamma =-5^{0}$, M=0, $W_{i}=0$, n=1.

Re	Skin friction at $\gamma =5^{0}$			Re	Skin friction at $\gamma =-5^{0}$		
	[45,46] Present				[37,45] Present		
20	−2.5271	−2.5271	−2.5269	10	−1.784547	−1.784547	−1.7845
60	− 3.9421	− 3.9421	− 3.9421	30	−1.413692	−1.413692	−1.4136
100	−5.8691	−5.8691	−5.8690	50	−1.121989	−1.121989	−1.1219
140	−8.2073	−8.2073	−8.2073	70	−0.893474	−0.893474	−0.8934
180	−10.7920	−10.7920	−10.7921	100	−0.640178	−0.640178	−0.6401

**Table 3:** Comparison of Nusselt number $-\frac{1}{\gamma }\theta^{'}\left(1\right)$ with previous results without nanofluid model Nb=0,Nt=0, for fixed $\gamma =-5^{0}$, $W_{i}=0$, n=1, $\delta_{E}=0$.

γ	[37]	[47]	[48]	Present study
${-5}^{0}$	0.04214811723	0.0421517243	0.0421520009	0.04215
$5^{0}$	0.03999321083	0.0399820121	0.0399841412	0.03998

## 7 Results and debates

The converging flow of a Carreau fluid past an inclined wedge is illustrated in this work. The heat and mass transport mechanisms are formulated using the Cattaneo-Christov theory. The two phase Buongiorno nanoparticles model and viscous heating are also included in the heat and mass equations, respectively. Previous investigation Graphics are used to obtain the numerical results to visualize the fluid's physical behavior. Each graph is composed of numerous curves, in which the red curves designate the diverging channel and blue curves indicate the converging channels. In the end, to analyze the role of Fourier's and non-Fourier's law during heat and mass transport a brief survey is presented in various tables.

### 7.1 Flow analysis

**Fig. 2:**
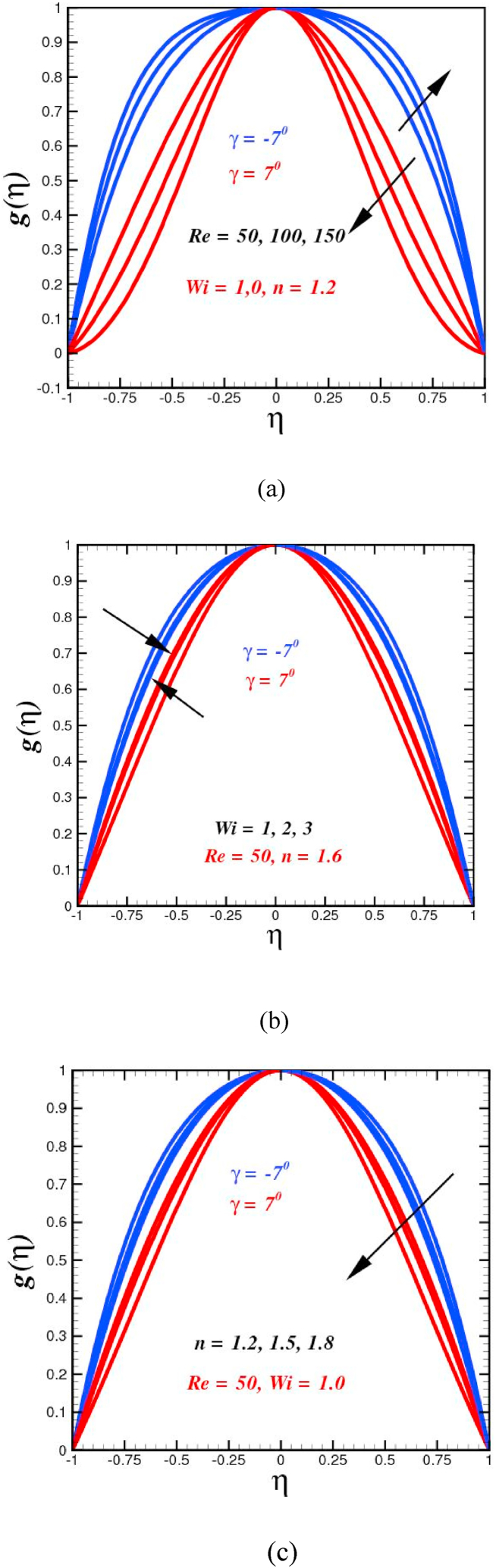
(a)Velocity curves g(η) for diverse numbers of Re. (b): Velocity curves g(η) for diverse numbers of Wi.(c): Velocity curves g(η) for diverse numbers of n.

**Fig. 3:**
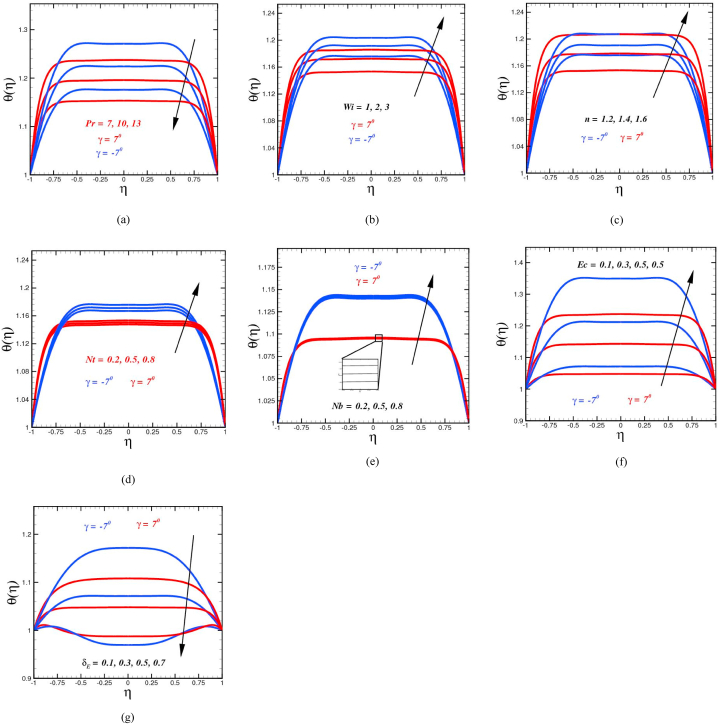
(a)Temperature curves θ(η) for diverse numbers of Pr. (b): Temperature curves θ(η) for diverse numbers of Wi.(c): Temperature curves θ(η) for diverse numbers of n. (d): Temperature curves θ(η) for diverse numbers of Nt. (e): Temperature curves θ(η) for diverse numbers of Nb.(f): Temperature curves θ(η) for diverse numbers of Ec. (g): Temperature curves θ(η) for diverse numbers of $\delta_{E}$.

The effects of Reynold number Re number on the Carreau fluid velocity g(η) in convergent diverging channel are depicted in Fig. 2(a). In fact, elevating Re number allows for the creation of a channel with a flatter profile in the middle and steep gradients along the walls. The thickness of the boundary layer therefore thins. It is abundantly evident that the backflow is completely prohibited in convergent flow circumstances. Fig. 3 illustrates the divergent flow, which concentrates volume flux at channels centers with lower gradients along the walls, is affected by Re number. The statistics show that the reversal of flow is substantially appreciated for just divergent channels. It is declared that the velocity profiles are symmetric when compared to η=0, and that symmetric converging flow is conceivable for a channel angle of 2γ that does not exceed $18^{0}$ degrees. It is also apparent that the backflow is diminished in the situation of a converging channel. On the other hand, the impact of the Re on diverging flow is to centralize the volume flux in the center of the channel with decreasing slopes near the walls, and as a result, the thickness of the momentum boundary layer improves as the Reynolds number upturns. For a purely diverging channel, symmetrical drift is not achievable for an apex angle of 2γ unless the Reynolds number does not reach a critical value. For a partially diverging channel, symmetric flow is conceivable for an opening angle of 2γ. Similar trend for flow domain due to variation of inertial forces was reported by Ref. [49]. As seen in Fig. 2(b), the Weissenberg number $W_{i}$ has an increasing influence on the diverging velocity near the channel wall, which enables more fluid to flow through the channel, but does not affect the velocity profile as the channel is moved farther away from the end of the channel. Converging velocity exhibits significantly diminishing tendency as $W_{i}$ increases in value. (See Fig. 2(b)). Physically, higher values of $W_{i}$ result in growth of momentum boundary layer thickness as result the narrowing channel velocity diminishes. The choice of Weissenberg number $W_{i}\left(=1,2,3\right)$ is chosen based on the similar approach was adopted by Carrington et al. [50]. The velocity sketch for improving 1<n<2 (shear-thickening) has de-escalating behavior as clear from Fig. 2(c). More resistance is faced for large values of less resistance is faced by the fluid. The fluid becomes thicker with an upsurge 1<n<2 , which leads to the velocity reduction. The fluid is characterized as shear-thinning 0<n<1 for, shear-thickening for n>1, and Newtonian for n=1. Implies that the Carreau liquid acts like a power-law fluid at high shear rates and a viscous fluid at low shear rates.

### 7.2 Thermal analysis

The temperature sketches θ(η) for distinct physical parameters are portrayed in Fig. 3. Fig. 3(a) demonstrates the features of the Prandtl number Pr on the temperature profile when a non-Fourier heat flow model is used in conjunction with the temperature profile. Temperature is a decreasing function of Pr. The elevation in the Prandtl number results in the reduction in the thickness of the thermal boundary layer. This value is derived from the correlation between momentum diffusivity and thermal diffusion. Concerning thermal and momentum boundary layers in heat transport problems, Pr regulates the relative thickness of these layers. When Pr, is small, heat diffuses fast in comparison to the velocity (momentum), which signifies that for fluid flow, the thickness of the thermal boundary layer is much greater than the thickness of the momentum boundary layer. As a result of their greater thermal conductivities (as well as thicker thermal boundary layer structures), fluids with fewer Prandtl numbers can conduct heat away from the sheet relatively quick than fluids with higher Prandtl numbers (reduced boundary layers). Therefore, the Prandtl number should be used to predict the amount of cooling in conducting flows when a conducting flow is being explored. Similar observation for Prandtl number were observed by Ref. [49]. Thermal distribution is directly associated with the Weissenberg number $W_{i}$ can be witnessed in Fig. 3(b). An augmentation in the Weissenberg number signifies an elevation in the relaxation time owing to which the energy profile is altered. Physically, it is the ratio between the shear rate to the relaxation time. As a result, with increased Weissenberg numbers, the fluid becomes thicker, and consequently, the velocity and the thickness of the layer simultaneously drop. Physically, uplifting Wi, the fluid nanoparticle offers more resistive force, consequently the thermal distribution upsurge. Augmentation in power index n, the temperature distribution upshot as prescribed in Fig. 3(c). The shear thickening fluid becomes more thickened on elevating power index n depressing the fluid movement and dominate the thermal distribution of the system. Growth in temperature against rising in n is witnessed in Fig. The mechanism of distribution of particles, in the existence of a temperature gradient, is known as thermophoresis. The overvaluation of Nt establishes a temperature gradient, which culminates in an increase in the force (thermophoretic) between nanoparticles because of the thermal distribution upsurge. This effect is crucial for stimulating the amount of fluid heated and raising the temperature as shown in Fig. 3(d). Brownian motion Nb(=0.2,0.5,0.8) is the chaotic migration of nanoparticles together within base fluid driven by the continuous collision of nanoparticles with the molecular of the working fluid. Brownian motion is a kind of movement of the particles as described in Fig. 3(e). With a growth in the number of encounters, heat transfer capabilities improve, and the value of temperature rises because of this increase. Brownian parameter Nb range is monitored within the range due to Ref. [51] results. Temperature fluctuation against altered values of Ec is presented in Fig. 3(f). According to Eckert number Ec, the relationship among stream kinetic energy and heat enthalpy difference. As a result, an augmentation in Eckert number results in a higher in kinetic energy. Furthermore, it is a well-known observation that temperature refer to the mean kinetic energy of a system. Thus, with alteration of Ec, the fluid temperature boosts. The temperature distribution vs the thermal time relaxation parameter $\delta_{E}$ is visualized in Fig. 3(g). The heat flux and the thickness of the corresponding boundary layer diminish as the value of $\delta_{E}$ increases. Physically, when the thermal time relaxation parameter $\delta_{E}$ is elevated, the fluid particles require extra time to transmit heat to the surrounding particles, causing a reduction in the temperature field. The classical Fourier's model is attained on setting $\delta_{E}=0$. In this scenario, infrared radiation is emitted across the material at an infinite rate, increasing temperature distribution when $\delta_{E}=0$.

### 7.3 Concentration analysis

**Fig. 4:**
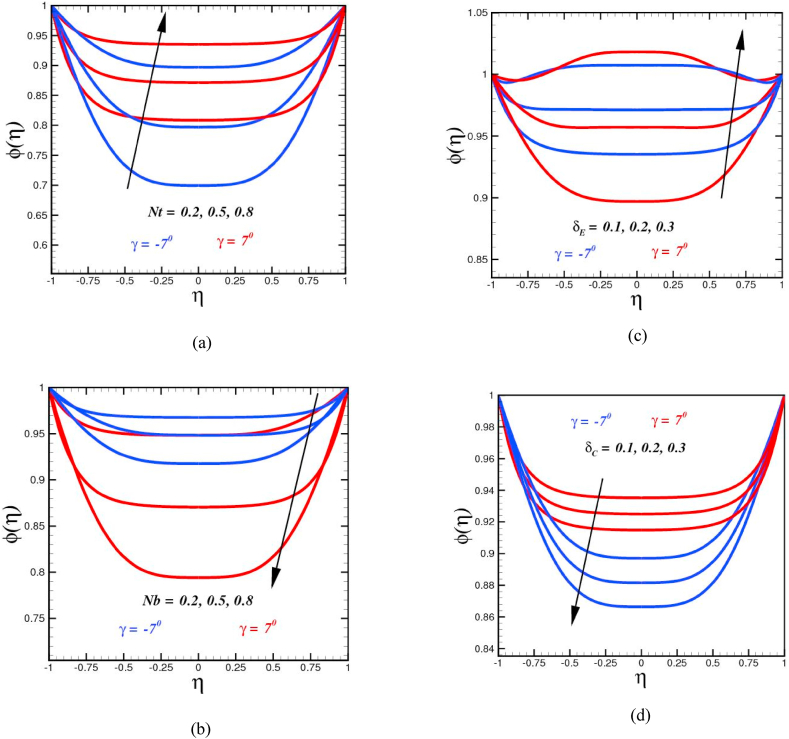
(a)Mass concentration curves φ(η) for diverse numbers of Nt.Fig. 4(b): Mass concentration curves φ(η) for diverse numbers of Nb.Fig. 4(c): Mass concentration curves φ(η) for diverse numbers of $\delta_{E}$.Fig. 4(d): Mass concentration curves φ(η) for diverse numbers of $\delta_{C}$.

Concentration sketches φ(η) for diverse parameters are displayed in Fig. 4. Concentration offers depressing behavior for higher thermophoretic parameter. It is shown that the mass distribution and the appropriate thickness of the concentration layer expand due to an augmentation in the thermophoretic parameter as the parameter advances as addressed in Fig. 4(a). Physically, increased values of the thermophoretic parameter Nt are corresponding to more thermophoretic forces which leads to more diffusive effects. The effects of the Brownian motion parameter Nb on the concentration profile are disclosed in Fig. 4(b). It has been shown that the fluid concentration falls with the growing significance of the Brownian motion parameter Nb. Higher values of Nb ultimately lead to a better number of collisions between the liquid particles, resulting in fewer mass transports and a reduction in the concentration field consequently. When the Brownian motion parameter is elevated, the boundary layer equivalent to the concentration field grows thinner. The action of thermal and solutal time relaxation parameter on concentration profile seems contrary as sketched in Fig. 4(c) and (d). Furthermore, Fig. 4(c), reveals that with improvement in thermal relaxation parameter, the concentration profile uplift. When the thermal time relaxation parameter $\delta_{E}$ is set to a high value, physically fluid particles entail extra time to transition heat to their nearby particles, resulting in an increase in the system's concentration. But on the other hand, with augmented value of the solutal relaxation time parameter $\delta_{C}$, particles need more time to diffuse, results in a diminishing of the concentration distribution as a consequence.

### 7.4 Assessments through tabulated values

A brief comparison for thermal and nanoparticle concentration when incorporating modified Fourier's law and Fick's law into energy and concentration equations with classical Fourier's heat flux model is prepared in Table 4, Table 5, Table 6, Table 7. The thermal and concentration distribution is evaluated keeping Pr=7 (water flow case). With fixed Pr=7, the thermal behavior significantly changes. Temperature profiles contain a lot of oscillations, including many maxima and minima. The heat transfer rate in the convergent channel is frequently influenced negatively by rising power-indexed n and the Weissenberg number $W_{i}$, but the Prandtl number and the Eckert number Ec escalates the thermal curves. The temperature appears to rise due to the thermophoresis parameter because of the thermal flux at the wall of the channel and the drift velocity produced by the integral particles towards the walls, but the haphazard motion of the nanoparticles tends to decrease the heat as they remove heat from the surface. The thermal curve in the divergent channel is negatively impacted by the fluid elasticity. Here, since the heat transport rate increases for both converging and divergent channels with rising Pr, it can be shown that fluids with higher Prandtl numbers function better as a heat transfer exponent. Higher Eckert numbers indicate increased heat over the surface due to greater viscosity, which is clearly shown in the table. Eckert number refers to the nanofluid capacity to dissipate heat. The chaotic mobility of the nanoparticles has a negative effect on the heat transfer rate in the converging channel but a significant improvement in the divergent channel. Thermophoretic behavior increases thermal efficiency in the converging channel but decreases it in the divergent channel.

**Table 4:** Temperature θ(η) distribution for non-Fourier's model when $\delta_{E}=0.2$, Re=50.

Pr	Nb	Nt	Ec	$W_{i}$	n	θ(η) $\gamma =-7^{0}$	θ(η) $\gamma =7^{0}$
7	0.4	0.2	0.1	1.0	1.1	1.0081	1.0046
	0.6					1.0089	1.0082
	0.8					1.0095	1.0140
7	0.4	0.2	0.1	1.0	1.1	1.0212	1.0079
		0.4				1.0217	1.0110
		0.6				1.0224	1.0174
7	0.4	0.2	0 1	1.0	1.1	1.0167	1.0249
			0.2			1.0176	1.0117
			0.3			1.0187	1.0221
7	0.4	0.2	0.1	1.0	1.1	1.0045	1.0016
				1.2		1.0204	1.0008
				1.4		1.0409	1.0028
7	0.4	0.2	0.1	1.0	1.1	1.0045	1.0105
					1.2	1.0204	1.0108
					1.3	1.0208	1.0064

**Table 5:** Temperature θ(η) distribution for classical Fourier's model when $\delta_{E}=0$, Re=50.

Pr	Nb	Nt	Ec	$W_{i}$	n	θ(η) $\gamma =-7^{0}$	θ(η) $\gamma =7^{0}$
7	0.4	0.2	0.1	1.0	1.1	1.0092	1.0058
	0.6					1.0052	1.0072
	0.8					1.0156	1.0094
7	0.4	0.2	0.1	1.0	1.1	1.0152	1.0018
		0.4				1.0166	1.0040
		0.6				1.0177	1.0062
7	0.4	0.2	0.1	1.0	1.1	1.0282	1.0121
			0.2			1.0312	1.0155
			0.3			1.0328	1.0208
7	0.4	0.2	0.1	1.0	1.1	1.0189	1.0180
				1.2		1.0219	1.0120
				1.4		1.0282	1.0120
7	0.4	0.2	0.1	1.0	1.1	1.0470	1.0299
					1.2	1.0296	1.0189
					1.3	1.0329	1.0201

**Table 6:** Concentration φ(η) distribution in the view of Fick's model when $\delta_{E}=0.2$, $\delta_{C}=0.1\text{,}$Re=50.

Pr	Nb	Nt	Ec	$W_{i}$	n	φ(η) $\gamma =-7^{0}$	φ(η) $\gamma =7^{0}$
7	0.4	0.2	0.1	1.0	1.1	0.9839	0.9894
	0.6					0.9746	0.9889
	0.8					0.9680	0.9884
7	0.4	0.2	0.1	1.0	1.1	0.9945	0.9965
		0.4				0.9951	0.9975
		0.6				0.9957	0.9985
7	0.4	0.2	0.1	1.0	1.1	0.9839	0.9894
			0.2			0.9844	0.9964
			0.3			0.9849	0.9963
7	0.4	0.2	0.1	1.0	1.1	0.9942	0.9988
				1.2		0.9982	0.9983
				1.4		0.9893	0.9993
7	0.4	0.2	0.1	1.0	1.1	0.9957	0.9965
					1.2	0.9835	0.9995
					1.3	0.9815	0.9980

**Table 7:** Concentration φ(η) distribution with classical Fourier's model where $\delta_{E}=0$, $\delta_{C}=0\text{,}$Re=50.

Pr	Nb	Nt	Ec	$W_{i}$	n	φ(η) $\gamma =-7^{0}$	φ(η) $\gamma =7^{0}$
7	0.4	0.2	0.1	1.0	1.1	0.9993	0.9995
	0.6					0.9992	0.9992
	0.8					0.9991	0.9989
7	0.4	0.2	0.1	1.0	1.1	0.9982	0.9980
		0.4				0.9985	0.9985
		0.6				0.9988	0.9990
7	0.4	0.2	0.1	1.0	1.1	0.9991	0.9980
			0.2			0.9992	0.9992
			0.2			0.9993	0.9997
7	0.4	0.2	0.1	1.0	1.1	0.9991	1.0000
				1.2		0.9994	0.9977
				1.4		0.9982	0.9995
7	0.4	0.2	0.1	1.0	1.1	0.9957	1.0000
					1.2	0.9991	0.9977
					1.2	0.9989	0.9972

## 8 Conclusion

This theoretical and computational work addresses the non-Newtonian nanofluids across an inclined channel with double diffusion Cattaneo- Christove theory. Instead of employing Fourier's and Fick's laws, the Cattaneo-Christov heat and mass flux theories are established to examine the heat and mass transport processes in the liquid. Constitutive equations are assembled in order model the relevant flow problem mathematically. These modeled partial differential equations are transformed into differential equations using a renovation procedure. Graphs and tables help to illustrate key findings. The uniqueness of the work is demonstrated by demonstrating how it is used in a diversity of industrial, technological, and liquid film in condensation processes, paper production, hot rolling, design of nozzles, converging dies, and plastic films drawing. Furthermore, this effort is novel and will certainly draw investigators because substantial efforts can be made in the future to inspect thermal and concentration fields in the context of classical Fourier's theory under a variety of physical parameters, including Brownian, thermophoresis, Eckert number, and many others. The major points from the investigation are mentioned below.

1. Improving the convergent flow inertial forces Re, a straighter profile can be created near the channel's center, which also leads to a narrower boundary layer.
2. Rising Re number causes the volume flux to distillate at the center of the channel in deviating flow. In these circumstances, the thickness of the fluid layer upturns by increasing the Re number.
3. Velocity streams shrink due to augmented power-indexed n, while the consequences of Weissenberg number $W_{i}$, in diverse geometry are diverse.
4. By include the thermal relaxation parameter, features of the Cattaneo-Christov heat flux model are observed. It is shown that the fluid's temperature lowers when the Cattaneo-Christov model is used.
5. The number of oscillations in diverging channels primarily depends on the type of fluid flowing through them. In fact, the oscillations are raised when the Prandtl number rises.
6. The progressive Brownian motion and thermophoresis of nanoparticle both depict opposite trend for concentration.

## Author contribution statement

Sohail Rehman: Performed the experiments; Wrote the paper.

Ayman Alfaleh; Hashim: Analyzed and interpreted the data.

Kallekh Afef: Contributed reagents, materials, analysis tools or data.

Syed Inayat Ali Shah: Conceived and designed the experiments.

## Data availability statement

Data included in article/supplementary material/referenced in article.

## Funding statement

The authors would like to thank the Deanship of Scientific Research at Umm-Al-Qura University for supporting this work by grant code: (22UQU4400074DSR02).

## Declaration of competing interest

Dear editor,

I hope you are fine and enjoying good health. I have no conflict of interest with any one.
